# Structural impairment in superficial and deep white matter in schizophrenia

**DOI:** 10.1017/neu.2023.44

**Published:** 2023-08-25

**Authors:** Sung Woo Joo, Young Tak Jo, Soojin Ahn, Young Jae Choi, Woohyeok Choi, Sang Kyoung Kim, Soohyun Joe, Jungsun Lee

**Affiliations:** 1 Department of Psychiatry, Asan Medical Center, University of Ulsan College of Medicine, Seoul, Republic of Korea; 2 Department of Psychiatry, Kangdong Sacred Heart Hospital, Hallym University College of Medicine, Seoul, Republic of Korea; 3 Brain Laboratory, Department of Psychiatry, University of California San Diego, School of Medicine, San Diego, CA, USA

**Keywords:** Diffusion magnetic resonance imaging, Schizophrenia, White mater, Anisotropy, Neuroimaging

## Abstract

**Objective:**

Although disconnectivity among brain regions has been one of the main hypotheses for schizophrenia, the superficial white matter (SWM) has received less attention in schizophrenia research than the deep white matter (DWM) owing to the challenge of consistent reconstruction across subjects.

**Methods:**

We obtained the diffusion magnetic resonance imaging (dMRI) data of 223 healthy controls and 143 patients with schizophrenia. After harmonising the raw dMRIs from three different studies, we performed whole-brain two-tensor tractography and fibre clustering on the tractography data. We compared the fractional anisotropy (FA) of white matter tracts between healthy controls and patients with schizophrenia. Spearman’s rho was adopted for the associations with clinical symptoms measured by the Positive and Negative Syndrome Scale (PANSS). The Bonferroni correction was used to adjust multiple testing.

**Results:**

Among the 33 DWM and 8 SWM tracts, patients with schizophrenia had a lower FA in 14 DWM and 4 SWM tracts than healthy controls, with small effect sizes. In the patient group, the FA deviations of the corticospinal and superficial–occipital tracts were negatively correlated with the PANSS negative score; however, this correlation was not evident after adjusting for multiple testing.

**Conclusion:**

We observed the structural impairments of both the DWM and SWM tracts in patients with schizophrenia. The SWM could be a potential target of interest in future research on neural biomarkers for schizophrenia.

Significant outcomes

- We investigated the structural impairments of both superficial and deep white matter tracts.
- We observed that patients with schizophrenia have a lower fractional anisotropy of several superficial and deep white matter tracts with small effect sizes.

Limitations

- The effects of medication were not considered in the analyses.
- The study has a cross-sectional design.

## Introduction

Disconnectivity among brain regions has been acknowledged as one of the main hypotheses for the neural mechanism behind schizophrenia (Friston *et al*., 2016). Diffusion magnetic resonance imaging (dMRI) studies have been conducted for the *in vivo* examination of structural abnormalities of white matter tracts in schizophrenia (Samartzis *et al*., 2014; Wheeler & Voineskos, 2014). White matter tracts, which are the anatomical structure connecting separate brain regions, can be divided into superficial white matter (SWM) and deep white matter (DWM) tracts according to their anatomical location. To date, most dMRI studies in schizophrenia research have focused on the structural impairments of DWM rather than those of SWM because of the challenges involved in the consistent reconstruction of SWM across subjects (Guevara *et al*., 2020). DWM can be consistently extracted across subjects compared with SWM because of its large and well-defined bundles. Despite the variability of previous results in terms of the location and magnitude of the structural impairments of DWM, the widespread structural abnormalities of DWM in schizophrenia have been consistently reported (Karlsgodt, 2016; Dietsche *et al*., 2017). According to Kelly et al., patients with schizophrenia have a lower fractional anisotropy (FA) in 20 major white matter tracts and that the largest effects sizes were observed in the anterior corona radiata and corpus callosum (Kelly *et al*., 2018).

Despite abundant evidence on the structural abnormalities of DWM, it is still uncertain how the clinical symptoms of schizophrenia, such as positive and negative symptoms and cognitive impairment, are associated with white matter abnormalities (Karlsgodt, 2016). Furthermore, existing literature lack consistency on the correlations between the FA of white matter tracts, severity of positive and negative symptoms, and cognitive impairment in patients with schizophrenia (Szeszko *et al*., 2008; Perez-Iglesias *et al*., 2010; Kochunov *et al*., 2017). Given that a mental process is operated by the simultaneous activation and deactivation of distinct brain regions, the SWM, which has been rarely included in studies, should be examined along with the DWM to reveal reliable neural biomarkers for schizophrenia.

The U-fibres or short association fibres of the SWM are located beneath the brain cortex and connect the adjacent gyri of the brain cortex. The large difference in SWM configuration across subjects is due to the variability of the cortical morphology, such as folding pattern and gyrification; this hampers the examination of the common structural abnormalities of the SWM across subjects and the anatomic labelling of structural abnormalities. The methods for identifying the SWM can be divided into ROI-based, fibre clustering and hybrid methods (Guevara *et al*., 2020). Fibre clustering methods are usually preferred in large-scale studies because of their automated process for the reconstruction and annotation of SWM; however, ROI-based methods have a higher extraction accuracy of the SWM at the individual level than fibre clustering methods. On the basis of the similarity among fibre clusters generated from tractography data, fibre clustering methods reconstruct and annotate SWM bundles via atlas-based schemes. Although there has been technical progress in the calculation of similarity among fibre clusters and the development of atlases for labelling SWM, the structural abnormalities of SWM associated with schizophrenia have been reported in only a few studies (Phillips *et al*., 2011; Nazeri *et al*., 2013; Ji *et al*., 2019). Given that it is one of the last parts of the brain to myelinate, the SWM has been reported to be vulnerable to many diseases, including schizophrenia (Phillips *et al*., 2016a; Phillips *et al*., 2016b; Duchatel *et al*., 2019). Although the SWM has a comparatively higher density of interstitial white matter neurons than other types of white matter, one of the robust SWM pathologies in patients with schizophrenia compared with healthy controls is the increased density of interstitial white matter neurons, which indicates cortical interneuron deficit in schizophrenia (Yang *et al*., 2011; Duchatel *et al*., 2019).

One of the critical issues in multisite neuroimaging studies is associated with the use of different scanners and image parameters across study sites, which can induce spurious findings because the biological variance of interest can be reduced or amplified by the scanner and/or image parameter-specific effects (Helmer *et al*., 2016). Previous multisite neuroimaging studies have adopted a statistical method that utilised the individual results from each study site and performed an analysis by combining them across study sites (Salimi-Khorshidi *et al*., 2009; Jahanshad *et al*., 2013; Kochunov *et al*., 2014). Despite the advantages of using standardised results from each study site, a notable caveat when using this method is that it cannot utilise the whole variance of the study population. A retrospective harmonisation method was developed on the basis of the raw dMRI data acquired from different study sites wherein different scanners and image parameters were used for image acquisition (Mirzaalian *et al*., 2018; Cetin-Karayumak *et al*., 2019). This registration-based harmonisation method creates templates by using the raw dMRI data of matched subsets from each study site and applies them to the raw dMRI data of subjects in the target sites. The harmonisation method can remove scanner-specific effects while preserving the biological variability of interest (Cetin-Karayumak *et al*., 2019). The notable advantage of this harmonisation method is that it enables researchers to utilise large sample size. Considering the small effect sizes reported in previous studies, sufficient statistical power should be obtained to reveal the group-level structural abnormalities of the brain in schizophrenia (Kelly *et al*., 2018).

This study aimed to investigate the structural abnormalities of the SWM and DWM in patients with schizophrenia by using publicly available neuroimaging datasets from several study projects. The retrospective harmonisation method was used to remove inter-project differences related to the use of different scanners and image parameters, thus allowing us to utilise a variance of the whole population included in this study. Our main hypothesis is that patients with schizophrenia have structural abnormalities in both the SWM and DWM compared with healthy controls. We performed correlation analyses for the structural abnormalities of the SWM and DWM associated with clinical symptoms as an exploratory approach.

## Material and methods

### Data collection and participants

We utilised three publicly available datasets from the Center of Biomedical Research Excellence (COBRE), Neuromorphometry by Computer Algorithm Chicago (NMorphCH) and University of California Los Angeles Consortium for Neuropsychiatric Phenomic LA5c Study (UCLA). The COBRE and NMorphCH datasets were obtained via SchizConnect (schizconnect.org) (Wang *et al*., 2016), and the UCLA dataset was obtained via OpenNeuro (openneuro.org) with accession number ds000030. These projects were aimed at elucidating the underlying neural mechanisms of psychiatric disorders, such as schizophrenia, by integrating various datasets from neuroimaging, neuropsychological tests and neurocognitive tasks. Further details on the projects have been described in previous studies (Alpert *et al*., 2016; Landis *et al*., 2016; Gorgolewski *et al*., 2017). We included patients who were strictly defined to have schizophrenia in the COBRE and NMorphCH datasets and excluded patients with schizoaffective disorder. For patients with schizophrenia in the NMorphCH and UCLA datasets, the clinical symptom severity was evaluated using the Scale for the Assessment of Positive Symptoms (SAPS) (Andreasen, 1984) and the Scale for the Assessment of Negative Symptoms (SANS) (Andreasen, 1989). We converted the SAPS and SANS scores into the positive and negative scores of the Positive and Negative Syndrome Scale (PANSS) by using the equations reported by van Erp *et al*. (2014).

All participants provided their written informed consent after a complete explanation of the study. The individual study projects included were approved by the local institutional review board (IRB) and were conducted according to the Declaration of Helsinki. This study was approved by the IRB of Asan Medical Center (IRB no. 2021-0423).

### Image processing

#### Data acquisition, quality control and preprocessing

Supplementary Table 1 shows the parameters on image acquisition. Although numerous institutions participated in each study project, the image parameters are the same within each study project. We visually inspected all of the T1-weighted images and dMRIs of the participants and excluded 13 participants because of poor image quality, such as signal dropouts and artefacts. Table 1 presents the demographic and clinical information of the participants. For the quality control of dMRIs, SlicerDiffusionQC (https://github.com/pnlbwh/SlicerDiffusionQC) software was used for the detection and removal of bad gradient volumes. After we confirmed the automatic classification results of good or bad gradient volumes by using SlicerDiffusionQC, the following preprocessing procedures were performed using the Psychiatry Neuroimaging Laboratory pipeline (https://github.com/pnlbwh/pnlpipe), which were axis alignment, centring, eddy current and head motion correction.

**Table 1.** Demographic and clinical characteristics of the participants

	COBRE		NMorphCH		UCLA		Total		Statistic		
	HC	SZ	HC	SZ	HC	SZ	HC	SZ	*t*/ χ^2^	df	*p*
Number of participants	74	58	36	40	113	45	223	143			
Age, mean (SD), year	38.2 (12.0)	39.0 (12.9)	31.3 (8.4)	32.7 (6.7)	31.6 (8.9)	35.9 (8.9)	33.8 (10.4)	36.3 (10.5)	−2.236	364	0.026
Male, *n* (%)	57 (77.0)	44 (75.9)	20 (55.6)	28 (70.0)	57 (50.4)	34 (75.6)	134 (60.1)	106 (74.1)	6.995	1	0.008
Right-handedness, *n* (%)	69 (93.2)	47 (81.0)	31 (86.1)	32 (80.0)	111 (98.2)	45 (100)	211 (94.6)	124 (86.7)	4.379	1	0.036
Age of onset, mean (SD), year		21.1 (8.4)		NA		NA					
Duration of illness, mean (SD), year		17.9 (13.1)		NA		NA					
Medication^a^											
Typical antipsychotic, *n* (%)		8 (14.0)		NA		3 (7.1)					
Atypical antipsychotic, *n* (%)		51 (89.5)		NA		41 (97.6)					
Olanzapine equivalent dose, mg/day		16.7 (13.0)		NA		23.2 (36.3)					
PANSS											
Total		65.5 (17.5)		NA		NA					
Positive		15.4 (5.2)		20.2 (6.8)		17.2 (4.6)					
Negative		17.8 (5.3)		21.8 (5.4)		16.6 (5.6)					
General		32.3 (8.1)		NA		NA					

NA, not available; HC, healthy control; SZ, schizophrenia; COBRE, Center of Biomedical Research Excellence; NMorphCH, Neuromorphometry by Computer Algorithm Chicago; UCLA, University of California Los Angeles Consortium for Neuropsychiatric Phenomic LA5c Study; SD, standard deviation; PANSS, Positive and Negative Syndrome Scale.

a Information on medications was available in 57 patients in COBRE and 42 patients in UCLA.

#### Harmonisation

We harmonised the raw dMRIs across the study projects by utilising the methods in a previous study (Cetin-Karayumak *et al*., 2019). The UCLA dataset was determined as the reference on the basis of the number of participants and image parameters. Twenty right-handed healthy controls were selected from each project and were matched for age and sex. Supplementary Table 2 shows the results for the validation of the matching procedure. We used dMRIharmonization software (https://github.com/pnlbwh/dMRIharmonization) for the later steps. To build scale maps generated from the pairs of rotation-invariant spherical harmonics feature templates, the preprocessed dMRIs of 20 healthy controls from each study project were used. The inter-project difference regarding the use of different scanners and image parameters was learned by building the scale maps. We then applied them to the preprocessed dMRIs of the participants in the target projects. The following default parameters were used for the harmonisation process: b-value: 1000 s/mm^2^; resample: 1.5 mm^3^; spherical harmonic order: 6; and number of zero-padding: 10. The validation of the harmonisation process was performed, with the mean FA value of whole-brain white matter skeleton calculated using the registered dMRIs in the Illinois Institute of Technology Human Brain Atlas (Zhang & Arfanakis, 2018). We performed an unpaired *t*-test to examine the difference in the mean FAs between the reference and target projects before and after the harmonisation. The harmonisation procedure decreased the difference in the mean FAs among the projects, and there was no significant difference in the mean FAs between the reference and target projects after the harmonisation (Supplementary Figure 1). For the later steps, we applied b-value mapping, resampling and Gibbs unringing to the preprocessed dMRIs of the subjects in the reference project.

#### Whole-brain tractography and identification of white matter tracts

Unscented Kalman filter-based two-tensor tractography was performed with the default options (Rathi *et al*., 2011) according to the standard pipeline (https://github.com/pnlbwh/pnlpipe). We parcellated the tractography data by using the O’Donnell Research Group (ORG) white matter atlas and whitematteranalysis (https://github.com/SlicerDMRI/whitematteranalysis) (Zhang *et al*., 2018). After the rigid-affine registration of the tractography data to the ORG atlas tractography data, fibre clustering of the registered tractography data was performed: (1) an initial 800-cluster white matter parcellation was created in accordance with the ORG atlas. (2) After the removal of false-positive clusters, the resulting fibre clusters were transformed into the input tractography space with information on the hemispheric location (left, right or commissural). (3) The fibre clusters were separated by their anatomical location. The fibre clusters were then appended to white matter tracts according to the anatomical definitions by the ORG atlas, resulting in 65 DWM tracts and 8 SWM fibres. The mean FA value of the white matter tracts was calculated using the FiberTractMeasurements module in 3D Slicer (https://slicer.org).

### Statistical analyses

All statistical analyses were performed using R software (ver. 4.0.2; R Development Core Team, Vienna, Austria). Statistical significance was determined on the basis of an α value of 0.05.

An unpaired *t*-test or chi-square test was used for the comparisons of demographic and clinical characteristics between the healthy controls and the patients with schizophrenia. We performed a linear regression analysis for the group-by-side interaction effects for the mean FA of white matter tracts located in both hemispheres; there were no significant group-by-side interaction effects (Supplementary Table 3). We averaged the mean FA of white matter tracts located in both hemispheres, thus resulting in a total of 41 white matter tracts (33 DWM tracts and 8 SWM fibres). For group comparisons of the mean FA value, we defined outliers as values below or above 1.5 times the interquartile range in each white matter tract. Analysis of covariance (ANCOVA) was performed with covariates of age, sex, age-by-sex interaction, age^2^ and age^2^-by-sex interaction. The Bonferroni correction was used to adjust a total of 41 multiple tests.

To evaluate the association with clinical symptoms, we calculated the FA deviation of white matter tracts in the patient group. The FA deviation was defined as a percent change of the mean FA value calculated by the difference between the predicted and real FA values. We estimated the predicted FA value by using an equation presented in a study reporting a change in the FA value of white matter tracts across the lifespan of patients with schizophrenia (Cetin-Karayumak *et al*., 2019):

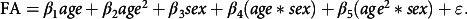




We created a prediction model by using the data of healthy controls and applied it to those of patients with schizophrenia to obtain the predicted FA value of each white matter tract. With regard to the association with clinical symptoms, we only included white matter tracts that had significant group differences in the mean FA. An exploratory correlation analysis was performed using Spearman’s rho, and the Bonferroni correction was applied to control multiple tests across the positive and negative PANSS scores and the number of included white matter tracts.

## Results

### Group comparisons of the FA of SWM and DWM

**Figure 1.**
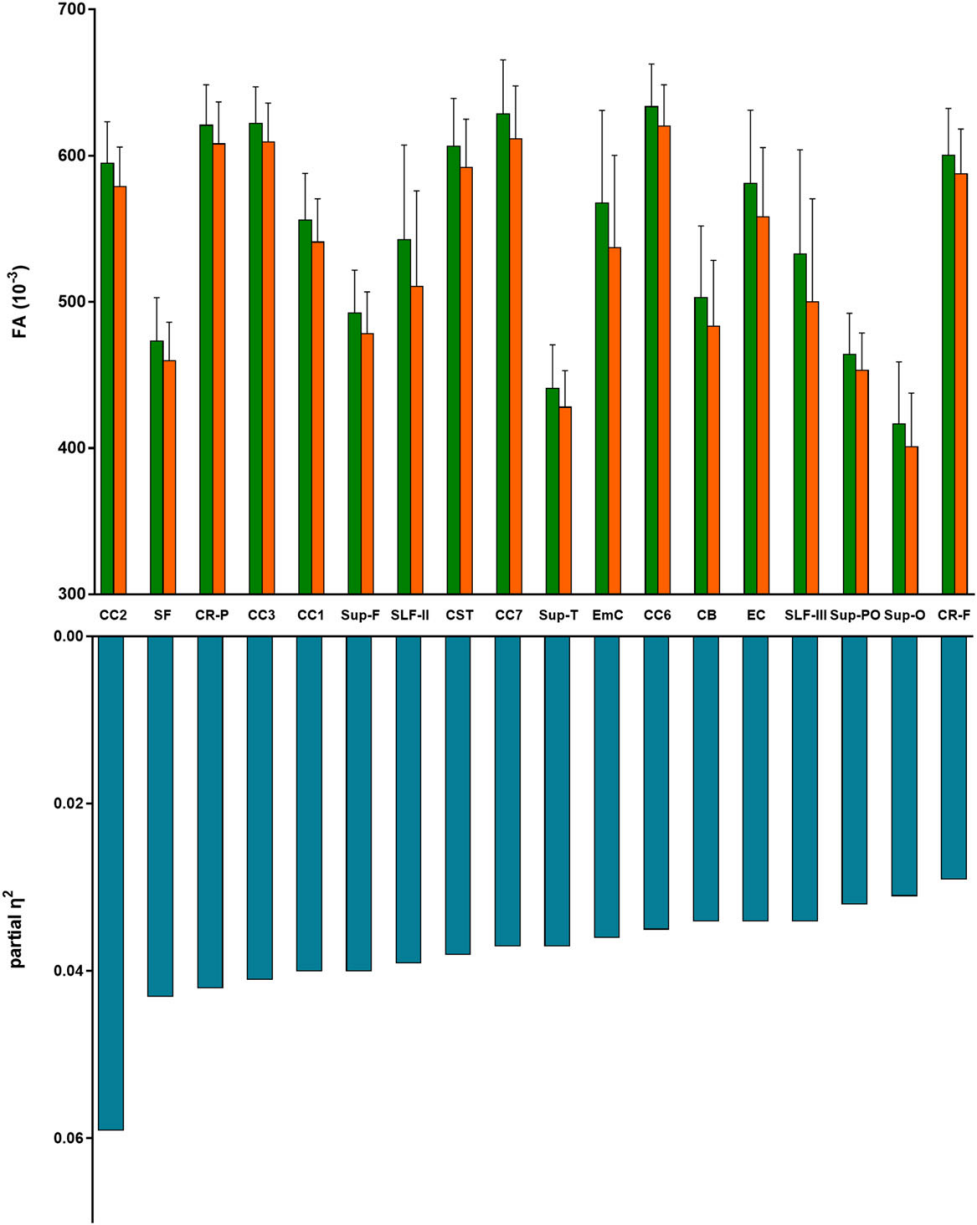
Fractional anisotropy (FA) and partial eta squared value of white matter fibres with a significant group difference in the mean FA between healthy controls and patients with schizophrenia. The mean and standard deviations of the FA values are presented in green bars (healthy controls) and orange bars (patients with schizophrenia). Blue bars show the partial eta squared value of the group difference. CB: cingulum bundle; CC: corpus callosum; CR-F: corona-radiata-frontal; CR-P: corona-radiata-parietal; CST: corticospinal tract; EC: external capsule; EmC: extreme capsule; SF: striato-frontal; SLF: superior longitudinal fasciculus; Sup-F: superficial–frontal; Sup-O: superficial–occipital; Sup-PO: superficial–parietal–occipital; Sup-T: superficial–temporal.

**Figure 2.**
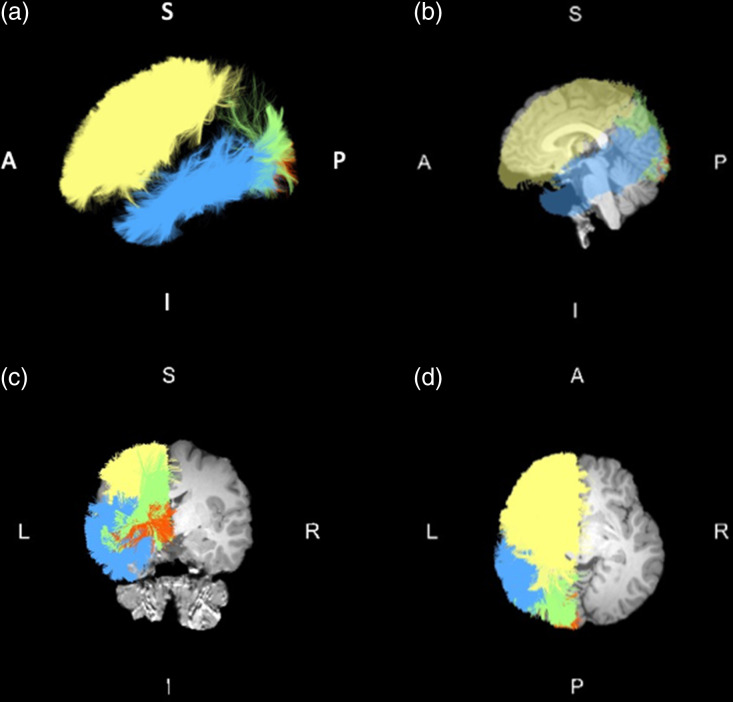
Example of superficial white matter tracts with significant group differences between patients with schizophrenia and healthy controls. Yellow, superficial–frontal; blue, superficial–temporal; orange, superficial–occipital; and green, superficial–parietal–occipital. (a) Left side, (b) sagittal view, (c) coronal view, and (d) axial view. Abbreviations: A (anterior), I (inferior), L (left), P (posterior), R (right), S (superior).

A total of 33 DWM and 8 SWM tracts were included in the group comparisons of the mean FA between healthy controls and patients with schizophrenia. ANCOVA with covariates of age, sex, age-by-sex interaction, age^2^ and age^2^-by-sex interaction revealed that 14 DWM tracts (CB, CC1, CC2, CC3, CC6, CC7, CR-F, CR-P, CST, EC, EmC, SF, SLF-II and SLF-III) and 4 SWM tracts (Sup-F, Sup-O, Sup-PO and Sup-T) had significant group differences after the Bonferroni correction for multiple comparisons. Figure 1 and Table 2 show the results of the group comparisons and abbreviations for the white matter tracts. All white matter tracts with significant group differences in the mean FA had small effect sizes according to Cohen’s criteria, thus indicating a partial eta squared value of 0.06 and 0.01 as the cut-offs for medium and small effect sizes, respectively (Cohen, 1988). Figure 2 illustrates superficial white matter tracts with significant group differences in FA between the two groups.

**Table 2.** Group comparisons of fractional anisotropy of superficial and deep white matter tracts^a^

Structure	Fractional anisotropy^b^, mean (SD)		*F*	df	*P* value for Bonferroni adjusted^c^	Partial η2
	HC	SZ				
AF	566.3 (75.5)	537.3 (74.1)	7.998	1357	0.203	0.021
CB	502.9 (49.1)	483.4 (45.1)	12.966	1357	0.015*	0.034
CC1	555.9 (32)	540.9 (29.6)	14.840	1350	0.006*	0.040
CC2	594.9 (28.3)	579 (26.9)	22.204	1350	<0.001**	0.059
CC3	622 (25.1)	609.3 (26.8)	15.530	1349	0.004*	0.041
CC4	629.1 (26.3)	618.2 (27.4)	10.244	1350	0.061	0.028
CC5	613.7 (31.6)	605.1 (32.9)	4.308	1349	1.000	0.012
CC6	633.5 (29.2)	620.3 (28.2)	12.769	1351	0.016*	0.035
CC7	628.6 (36.9)	611.4 (36.3)	13.841	1352	0.009*	0.037
CPC	603.7 (29.2)	595.4 (31.1)	3.453	1339	1.000	0.010
CR-F	600.2 (32.1)	587.5 (30.8)	10.655	1351	0.049*	0.029
CR-P	620.8 (27.7)	608.1 (28.7)	15.440	1350	0.004*	0.042
CST	606.3 (32.9)	592 (33)	14.050	1352	0.009*	0.038
EC	581 (50.1)	558.3 (47.2)	13.159	1352	0.013*	0.034
EmC	567.4 (63.5)	537 (63.2)	13.714	1356	0.010*	0.036
ICP	464.3 (51.4)	452.9 (49.2)	2.407	1330	1.000	0.007
ILF	519.2 (58.8)	495 (57.2)	9.711	1357	0.081	0.026
Intra-CBLM-I&P	382.6 (26.9)	383.4 (21.5)	0.168	1342	1.000	0.000
Intra-CBLM-PaT	264.3 (19.3)	257.5 (19.6)	8.949	1343	0.122	0.025
IOFF	598.5 (86.1)	563.1 (80.4)	9.375	1356	0.097	0.025
MCP	582.4 (44.5)	578.9 (43.9)	0.110	1338	1.000	0.000
MdLF	548.6 (41.6)	534.9 (36.6)	6.567	1357	0.443	0.018
PLIC	566.4 (25.4)	557.6 (25.1)	9.372	1353	0.097	0.025
SF	473.3 (29.6)	459.6 (26.5)	16.262	1354	0.003*	0.043
SLF-I	487.6 (32.6)	479 (33.7)	6.135	1355	0.562	0.016
SLF-II	542.6 (64.7)	510.7 (65.2)	15.006	1357	0.005*	0.039
SLF-III	532.8 (71.2)	500.1 (70.4)	13.150	1357	0.014*	0.034
SO	557 (38.9)	547 (40.9)	3.550	1347	1.000	0.010
SP	532 (32.5)	524.8 (32.9)	3.615	1356	1.000	0.010
Sup-F	492.4 (29.3)	478.4 (28.5)	15.328	1353	0.004*	0.040
Sup-FP	490.9 (42.2)	477.1 (41.2)	6.720	1357	0.407	0.018
Sup-O	416.7 (42.3)	401 (36.6)	11.475	1355	0.032*	0.031
Sup-OT	513.5 (29.6)	501.8 (28.6)	9.330	1345	0.100	0.026
Sup-P	487.3 (36.7)	473.3 (34.8)	8.883	1357	0.126	0.024
Sup-PO	464 (28.2)	453.4 (25.4)	12.027	1351	0.024*	0.032
Sup-PT	504.2 (36.4)	492.3 (35.2)	5.777	1354	0.687	0.016
Sup-T	440.8 (29.8)	428 (25)	14.124	1356	0.008*	0.037
TF	511 (37.6)	496.9 (31.6)	10.141	1357	0.065	0.027
TO	549 (44.1)	532.2 (40.8)	9.708	1353	0.081	0.026
TP	522 (31.3)	511.1 (28.6)	8.597	1357	0.147	0.023
UF	480.2 (31.4)	471.3 (31.4)	3.854	1349	1.000	0.011

SD, standard deviation; AF, arcuate fasciculus; CB, cingulum bundle; CC, corpus callosum; CPC, cortico-ponto-cerebellar; CR-F, corona-radiata-frontal; CR-P, corona-radiata-parietal; CST, corticospinal tract; EC, external capsule; EmC, extreme capsule; ICP, inferior cerebellar peduncle; ILF, inferior longitudinal fasciculus; Intra-CBLM-I&P, intracerebellar input and Purkinje tract; Intra-CBLM-PaT, intracerebellar parallel tract; IOFF, inferior occipitofrontal fasciculus; MCP, middle cerebellar peduncle; MdLF, middle longitudinal fasciculus; PLIC, posterior limb of internal capsule; SF, striato-frontal; SLF, superior longitudinal fasciculus; SO, striato-occipital; SP, striato-parietal; Sup-F, superficial-frontal; Sup-FP, superficial-frontal-parietal; Sup-O, superficial-occipital; Sup-P, superficial-parietal; Sup-PO, superficial-parietal-occipital; Sup-PT, superficial-parietal-temporal; Sup-T, superficial-temporal; TF, thalamo-frontal; TO, thalamo-occipital; TP, thalamo-parietal; UF, uncinate fasciculus; HC, healthy controls; SZ, schizophrenia.

a Analysis of covariance was performed, covarying of age, sex, age-by-sex, age^2^ and age^2^-by-sex.

b Presented as values * 10^−3^.

c *p < 0.05, **p < 0.001.

### Association of FA deviation with clinical symptoms

We performed exploratory correlation analyses of FA deviations with positive and negative PANSS scores in the patient group by using Spearmen’s rho (Table 3). The negative PANSS scores were negatively correlated with the FA deviations of the corticospinal tract (rho = −0.167, *p* = 0.048) and superficial–occipital tract (rho = −0.187, *p* = 0.027). However, these correlations did not persist after adjusting for multiple testing.

**Table 3.** Correlation of fractional anisotropy deviations of white matter tracts with clinical symptoms

Structure	PANSS positive		PANSS negative	
	rho	Unadjusted *p*	rho	Unadjusted *p*
CB	0.118	0.164	−0.054	0.523
CC1	0.006	0.941	−0.059	0.486
CC2	0.023	0.786	−0.071	0.400
CC3	0.091	0.283	−0.047	0.582
CC6	0.111	0.193	−0.096	0.258
CC7	−0.004	0.966	−0.15	0.076
CR-F	0.031	0.716	−0.152	0.072
CR-P	0.032	0.708	−0.124	0.146
CST	0.016	0.847	−0.167	0.048
EC	0.068	0.429	−0.124	0.145
EmC	0.004	0.965	−0.152	0.072
SF	0.03	0.721	−0.091	0.284
SLF-III	0.094	0.268	−0.095	0.263
SLF-II	0.079	0.352	−0.101	0.232
Sup-F	0.104	0.221	−0.092	0.280
Sup-O	−0.051	0.547	−0.187	0.027
Sup-PO	−0.03	0.725	−0.148	0.082
Sup-T	0.085	0.317	−0.09	0.289

CB, cingulum bundle; CC, corpus callosum; CR-F, corona-radiata-frontal; CR-P, corona-radiata-parietal; CST, corticospinal tract; EC, external capsule; EmC, extreme capsule; SF, striato-frontal; SLF, superior longitudinal fasciculus; Sup-F, superficial-frontal; Sup-O, superficial-occipital; Sup-PO, superficial-parietal-occipital; Sup-T, superficial-temporal; PANSS, Positive and Negative Syndrome Scale.

## Discussion

We investigated the structural abnormalities of the SWM and DWM tracts in patients with schizophrenia by using whole-brain two-tensor tractography and the fibre clustering method. Lower FA values in patients with schizophrenia were observed in 14 DWM and 4 SWM tracts after the Bonferroni correction for multiple comparisons. The negative PANSS scores in the patient group were negatively correlated with the FA deviations of the corticospinal and superficial–occipital tracts; however, this correlation did not persist after adjusting for multiple testing.

We found a lower FA value in the superficial–frontal, occipital, parietal–occipital and temporal tracts in patients with schizophrenia compared to that in healthy controls. Our results reflected those of studies on SWM abnormalities in schizophrenia. In comparing patients with schizophrenia and healthy controls, Phillips *et al*. found a reduced FA of SWM in the left temporal and bilateral occipital regions (Phillips *et al*., 2011). Nazeri *et al*. found a decreased FA in five SWM clusters located in the left posterior parietal–occipital and frontal regions (Nazeri *et al*., 2013). A recent study by Ji *et al*. found an increase or decrease in the generalised FA of the SWM tracts of patients with schizophrenia compared with that of healthy controls (Ji *et al*., 2019). Although a lower generalised FA value of SWM tracts was observed in the frontal, parietal and temporal regions than in other regions, the generalised FA of SWM tracts associated with the default mode network was increased. Our results add to findings regarding the disrupted SWM fibres in schizophrenia, thus emphasising the importance of SWM in the investigation for a better biological understanding of schizophrenia.

Similar to studies reporting DWM abnormalities in schizophrenia (Samartzis *et al*., 2014; Wheeler & Voineskos, 2014; Karlsgodt, 2016), we found that patients with schizophrenia had a reduced FA in several DWM tracts across the association, projection and commissural tracts. All white matter tracts with significant group differences in the mean FA had small effect sizes, and the greatest effect was observed in the genu of the corpus callosum. This result aligned with the findings of Kelly *et al*. (2018). We also observed a lower FA in the striato-frontal tracts (Oh *et al*., 2009; Quan *et al*., 2013; Levitt *et al*., 2017), posterior part of the corona radiata (Kelly *et al*., 2018), superior longitudinal fasciculus (Karlsgodt *et al*., 2008; Rowland *et al*., 2009; Szeszko *et al*., 2018) and cingulum bundle (Whitford *et al*., 2014; Whitford *et al*., 2015) in patients with schizophrenia. This finding has been consistently reported in previous research.

We used FA deviation instead of an absolute FA value to evaluate associations with clinical symptoms in the patient group. Given the effects of healthy ageing (Cetin-Karayumak *et al*., 2019) and sexual dimorphism (Lang *et al*., 2018) on FA, it seemed that FA deviation had an advantage over the absolute FA value in terms of reflecting the disease-specific effect. However, our correlation analyses showed that FA deviations had no significant associations with clinical symptoms. This negative finding could be attributed to the following: the patient group in our study showed a limited variation in the severity of clinical symptoms, and correlation analysis would be insufficient in capturing the association with clinical symptoms. Future studies with larger patient populations, heterogeneous clinical characteristics and sophisticated methodology are required to address this issue.

Our findings should be interpreted with caution because of the following limitations. First, the effect of medications was not considered because information on medications was missing in some datasets. Furthermore, existing results had conflicting information regarding the effect of medications on the FA value of white matter tracts. Although some studies have reported the significant effects of antipsychotics on the diffusion measures of white matter tracts (Szeszko *et al*., 2014; Xiao *et al*., 2018), other studies have not (Mamah *et al*., 2019; Koshiyama *et al*., 2020). Whether the long-term exposure of antipsychotics is associated with the structural change of white matter tracts and whether the effect of antipsychotics is differentiated by the type of antipsychotics is still an area of open discussion. Second, the cross-sectional design of the present study limited the establishment of causal relationships. Our results should be interpreted with the inherent limitations of a cross-sectional design. Future longitudinal studies are needed to investigate whether the structural abnormalities of the SWM and WMM tracts can aid in predicting the severity of clinical symptoms in patients with schizophrenia. Third, although we used FA deviation to control the confounding effects of age and sex on the FA values of white matter tracts, the differential effects of age and sex on each white matter tract have been reported (Cox *et al*., 2016; Cetin-Karayumak *et al*., 2020). Despite previous results on the effects of ethnicity and socio-economic status on white matter tracts (Brickman *et al*., 2008; Shaked *et al*., 2019), we did not consider these factors because of limited information. Further studies with advanced methods for adjusting the confounding effects are needed to compensate for this limitation of the current study. Fourth, informed consent is required for this type of neuroimaging study, and this requirement may be associated with an inherent limitation in excluding patients with severe clinical symptoms and not capturing the entire study population of interest. A more comprehensive neuroimaging dataset in terms of clinical symptoms would be beneficial to reveal the associations of FA values with clinical symptoms. Fifth, the results on the structural abnormalities of white matter tracts could not indicate a specific pathology to schizophrenia because we did not include patients with other psychiatric disorders. Comparisons between patients with schizophrenia and those with other psychiatric disorders would aid in examining whether the current results may be specific to schizophrenia.

We found that patients with schizophrenia had a lower FA value in several SWM and DWM tracts and that all white matter tracts with significant group differences in the mean FA had small effect sizes. The large sample size generated from the retrospective harmonisation method provided a notable advantage in revealing the structural abnormalities of the SWM and DWM tracts compared with similar previous studies. Our results contribute to improving the current understanding of the neural mechanisms of schizophrenia. Further research is needed to enhance our biological understanding and eventually aid in the recovery of patients with schizophrenia from the disease.

## Supplementary material

The supplementary material for this article can be found at https://doi.org/10.1017/neu.2023.44
